# Improving the thermoelectric power factor of PEDOT:PSS with 4,4'-bipyridine and LiBF _4_

**DOI:** 10.12688/openreseurope.20654.1

**Published:** 2025-08-04

**Authors:** Md Mahmudur Rahman, Mauricio Solis-de la Fuente, Lourdes Márquez-García, Sergio Castro-Ruiz, Estelle Liautaud, Lucie Fournier, Camille Chatard, Agathe Bouvet-Marchand, Mario Culebras, Jorge García-Cañadas

**Affiliations:** 1 Department of Industrial Systems Engineering and Design, Universitat Jaume I, Castelló de la Plana, Valencian Community, 12006, Spain; 2 Specific Polymers, Castries, 34160, France; 3 Institute of Material Science, University of Valencia, Valencia, 46071, Spain

**Keywords:** thin film, organic thermoelectric, electrolyte, thermoelectric polymer, viologen, impedance spectroscopy.

## Abstract

**Background:**

Thermoelectric (TE) materials can directly convert heat into electricity, which is beneficial for energy sustainability. Organic conducting polymers are TE materials that have drawn significant attention owing to different favorable properties, such as good processability, availability, flexibility, and intrinsically low thermal conductivity. Among the organic TEs, poly(3,4-ethylenedioxythiophene):polystyrene sulfonate (PEDOT:PSS) is the most extensively investigated material because of its stability and high electrical conductivity. The power factor ( *PF*) of PEDOT:PSS can be increased using different strategies, such as secondary doping, dedoping, energy filtering, and sequential post-treatments. All these strategies involve the contact of the polymer with different compounds.

**Methods:**

Herein, we have analyzed the impact on the *PF* of the treatment of PEDOT:PSS with two different systems: (i) a 0.1M solution of 4,4'-bipyridine in 3-methoxypropionitrile and (ii) a 0.1M solution of LiBF _4_ in the same solvent. Impedance, Raman, and ultraviolet-visible-near infrared spectroscopies were employed to understand the variations observed.

**Results:**

The results show that after the treatments, the Seebeck coefficient increased from ca. 12 to ca. 21 μV/K in both cases, and the electrical resistance of the film increased by 46.78% for 4,4'-bipyridine, and only 4.38% in the case of LiBF _4_, reaching at least 2.08 and 3.53 times *PF* improvements, respectively, with respect to the initial *PF* value (6.32 μWK ^-2^m ^-1^). The impedance spectroscopy analysis revealed that only an ohmic behavior existed in all cases. In addition, Raman and UV-vis-NIR analyses identified a dedoping mechanism, which explains the Seebeck coefficient variations identified in both treatments and the increase in electrical resistance for 4,4'-bypiridine. The remarkable lack of resistance increase for LiBF _4_ points to a different phenomenon that could be related to morphological effects.

**Conclusion:**

These two new treatments demonstrate their capability to reach *PF* values close to the state of the art and expand the catalogue of treatments available for PEDOT:PSS.

## Introduction

Approximately 72% of the global energy use is lost as waste heat ^1^. Remarkably, only a 10% recovery of this heat energy would surpass the total heat generated by modern sustainable energy sources (solar, wind, geothermal, and hydro-energy) ^2, 3^. In addition to waste heat, abundant thermal energy sources, such as the sun or even human bodies, are commonly available. Hence, technologies capable of converting heat into electricity are highly desirable, and can potentially contribute to reducing the current energy crisis. Thermoelectric (TE) devices are one of these technologies. They have the ability to convert heat into electricity, and vice versa, in a clean, environmentally friendly, and silent manner ^4, 5^. This technology has already been applied for various purposes such as power generation in car exhausts, space exploration, industrial furnaces, solar power generation, and sensors, among many others ^4– 6^.

Organic TE materials constitute an important family within TEs. The most studied compound in this family is the conducting polymer poly(3,4-ethylenedioxythiophene):polystyrene sulfonate (PEDOT:PSS), mainly because of its favorable properties such as easy processability, environmental stability, availability, flexibility, and intrinsic low thermal conductivity ^7^. PEDOT:PSS is typically formed by positively charged PEDOT and negatively charged PSS, which serve as counterions for charge balance and allow the dispersion of PEDOT in aqueous media. Usually, the as-prepared PEDOT:PSS shows low electrical conductivity *σ* (0.1–10 S/cm) ^8, 9^ as well as a low Seebeck coefficient *S* (15–18 μV/K) ^10^.

To improve the TE properties of PEDOT:PSS, various strategies, such as secondary doping, dedoping, post-solvent treatment, and energy filtering, have been employed ^11– 13^. Secondary doping increases *σ* by inducing changes in the molecular conformation and crystallinity of conducting polymer ^14^.

Dedoping is a strategy to obtain high power factors ( *PF*= *S* ^2^ *σ*) by reducing the doping level in PEDOT:PSS; hence, increasing *S* significantly with the common reduction of *σ*, reaching an overall *PF* improvement. Dedoping can be achieved using either a reducing agent or an acid-base treatment ^7, 15, 16^.

Energy filtering is another method to increase *S*. This strategy is based on the creation of barriers at the interface between the electronic polymer matrix and the fillers. These barriers block the transport of low-energy carriers, which increases the mean kinetic energy of the charge carriers, and hence, *S* increases ^12^.

Post-treatments are another way to improve the TE properties of PEDOT:PSS and can produce a simultaneous increase in *S* and *σ* by performing sequential treatments. The first treatment usually involves the *σ* increase, whereas the sequential treatment increases *S* by reducing the oxidation level ^17, 18^.

In this study, we investigated two new treatments to obtain *PF* improvements in PEDOT:PSS. The compounds used in the treatments were (i) a 0.1 M solution of LiBF _4_ in 3-methoxipropionitrile (3-MPN) and (ii) a 0.1 M solution of 4.4´-bipyridine also in 3-MPN. The improvements achieved were investigated by impedance spectroscopy and ultraviolet-visible-near infrared (UV-vis-NIR) and Raman spectroscopy, which revealed different changes produced in the films able to explain the *PF* variations found.

## Methods

**Figure 1.**
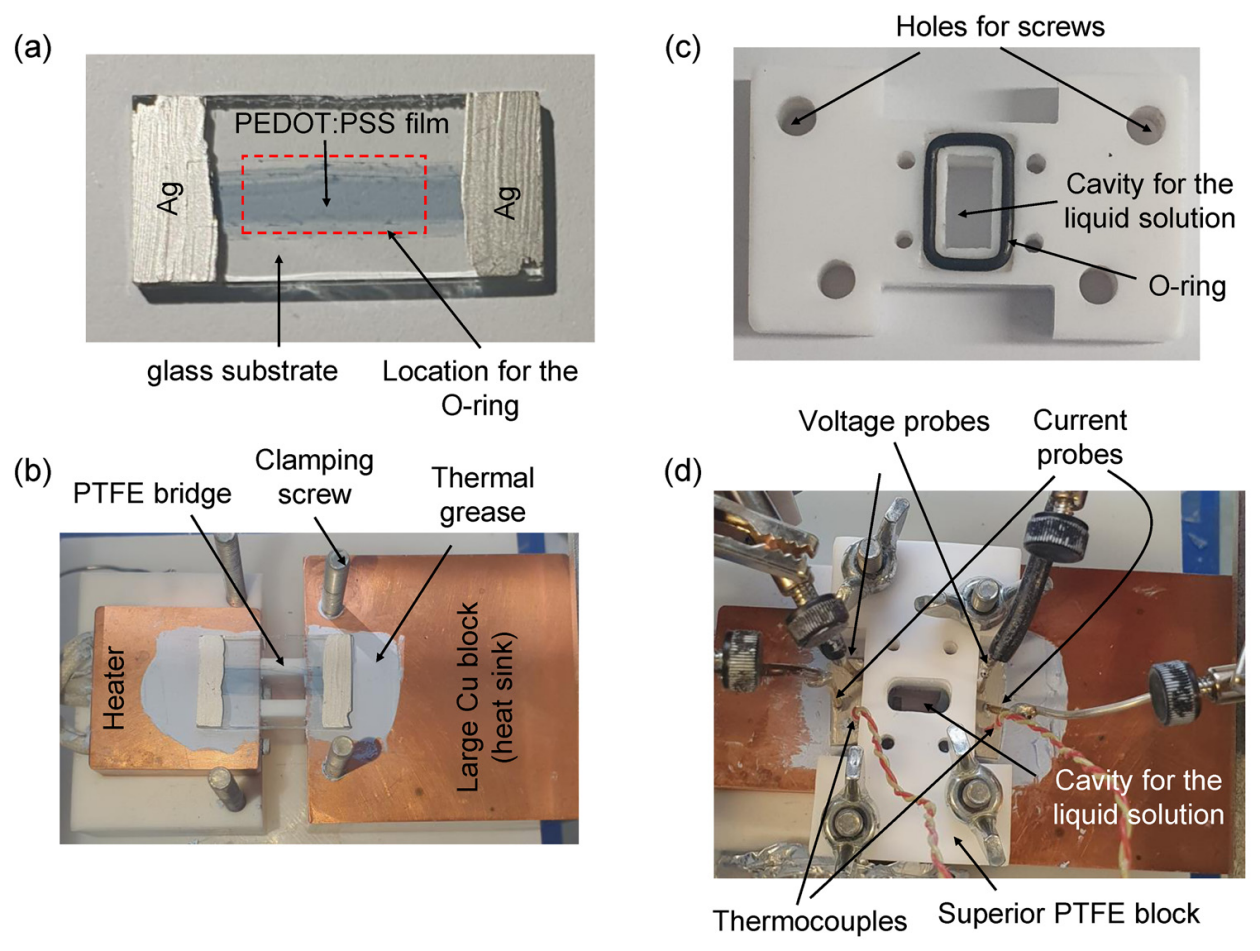
Cell and setup. Photographs of ( **a**) the prepared film with Ag paint contacts, ( **b**) the TE measurement setup without the PTFE block, ( **c**) the bottom part of the PTFE block, and ( **d**) the complete setup assembled.

9.417 g of PEDOT:PSS aqueous solution (Clevios PH1000, Ossila) was combined with 0.036 g of polyethyleneglycol (Pluriol E4005, BASF) and 0.547 g of dimethylsulfoxide (Carlo Erba, ref. 445103) to prepare different films. PEDOT:PSS films were deposited on soda-lime glass substrates of 25 mm × 15 mm area and 1.8 mm thickness. The glasses were cleaned before film deposition by means of three sonication steps of 15 min in different media. In the first step, sonication was performed using a soap (Labkem, SOAP-0685K0)/water solution (1:10 v/v). Then, in the second step, distilled water was used to remove excess soap. Finally, isopropanol (Labkem, PROL-P0P-5K0) medium was employed for sonication. After this, substrates were dried under a compressed air flow and treated in a UV ozone cleaner (Ossila, L2002A2-UK) for 20 min. Four layers of the PEDOT:PSS formulation (40 μL/layer) were deposited by spin coating at 500 rpm for 30 s, and then at 5000 rpm for 30 s. Before carrying out the spin coating, a Kapton tape mask was applied on the glass substrate to limit the deposition to a centered area of the substrate of 22 mm × 5 mm. After the deposition of each layer, the films were heated on a hot plate at 120°C for 10 min and subsequently cooled down in air for 10 min. Finally, the films were heated on a hot plate at 120°C for 30 min. The prepared films were then contacted at their ends using Ag paint (RS, ref. 186-3600), as shown in Figure 1a.

The Seebeck coefficient and electrical resistance measurements of the prepared films were carried out in a homemade setup, where two copper blocks were used to create a temperature difference Δ *T* (see Figure 1b). One of them, with 30 mm × 30 mm × 10 mm dimensions and three cartridge heaters inserted (Watlow, ref. C1E13-L12), was used as heat source. The other block, with dimensions of 50 mm × 50 mm × 30 mm, served as a heat sink. The two copper blocks were separated by a polytetrafluoroethylene (PTFE) bridge, which was used to relieve tension in the glass due to the pressure applied with an O-ring. The prepared films were placed on top of the copper blocks, as shown in Figure 1b. Thermal grease (RS, ref. 2173835) was applied at the glass/copper interfaces to improve thermal contacts. To accommodate the different solutions (4,4'-bypiridine and LiBF _4_) on top of the film, the bottom part of a holed PTFE block with a rectangular O-ring was placed on top of the glass substrate (see Figure 1c). The dashed line in Figure 1a corresponds to the position of the O-ring, which establishes the area of the film in contact with the solution. It should be noted that the pressure of the O-ring was held on the film and not on the Ag contacts. The PTFE block has also 4 screws, which provided the gentle pressure needed for sealing and prevented the solution from escaping.

To measure the Seebeck coefficient, two K-type thermocouples (RS, ref. 8140134) were placed on top of the Ag paint near the ends of the film (see Figure 1d). A small amount of thermal grease was used on the tip of the thermocouples to improve thermal contacts. The open-circuit voltage *V _oc_* was measured using two spring probes (RS, ref. 2615092) positioned on top of the Ag paint. The Seebeck coefficient was measured using the slope of the *V _oc_* versus the temperature difference. A nanovoltmeter (Keithley 2182A) was employed to measure the *V _oc_*, and the Δ *T* was recorded using a dual datalogger thermometer (RS, ref. 1316). The error in the Seebeck coefficient measurements, calculated from the linear fitting error, was below 3%.

The electrical resistance *R* of the films was measured in a pseudo 4-probe mode, using the previously mentioned probes for the voltage, and two similar probes contacted on the Ag paint contacts for the current *I*. The electrical resistance was then obtained from the slope of current-voltage ( *I-V*) curves, performed at Δ *T*=0 K with a Keithley 2450 source meter using a current scan with a 1 ms delay time. The error in the electrical resistance measurements, calculated from the linear fitting error, was below 1%.

The prepared films were treated with two solutions: (i) 0.1 M LiBF _4_ (Sigma Aldrich, ref. 244767) in 3-MPN (Sigma Aldrich, ref. 65290) and (ii) 0.1 M 4.4´-bipyridine (Sigma Aldrich, ref. S8178858148) also in 3-MPN. The sequence of measurements and the treatment adopted for each solution were as follows: first, the Seebeck coefficient and electrical resistance of the as-prepared sample were measured. Then, the solution (270 μL) was added to the cavity of the top PTFE block (see Figure 1d) and left for 2 h. After this, the solution was removed with a pipette, and the film was left to dry overnight. Finally, the Seebeck coefficient and the electrical resistance were measured again. It should be noted that the probes and thermocouples were not moved between measurements to obtain a more precise comparison.

To determine the electrical conductivity of the films, the thickness *t* of one of them was measured by profilometry (Sensofar PLμ2300), along with its resistance (as described above), width *W* and length (distance between voltage contacts) *L*. This formula was used to calculate the electrical conductivity *σ*= *L*/( *RWt*).

Different UV-vis-NIR absorption spectra of the PEDOT:PSS films were obtained using a PerkinElmer Lambda 1050 spectrometer. In addition, Raman spectroscopy measurements were performed using a Jasco NRS-3100 spectrometer equipped with an optical microscope and an air-cooled CCD detector.

Impedance spectroscopy measurements were carried out at Δ *T*=5 K for the films before and after the treatment using the same configuration of the probes that were employed to determine the electrical resistance. Measurements were performed in potentiostatic mode at 0 V dc current in the 10 mHz-1 kHz frequency range with an amplitude of 50 mV. A Metrohm Autolab PGSTAT204 instrument equipped with a FRA32M frequency response analyzer was used.

## Results and discussion

To obtain the initial *PF* of the prepared films, we measured the Seebeck coefficient and electrical conductivity of the film whose thickness was measured. The thickness, width and length obtained were 0.24 μm, 3.6 mm and 24.5 mm, respectively. On the other hand, the electrical resistance of the film was 0.58 kΩ, thus, *σ*=488.90 Scm ^-1^, and the Seebeck coefficient was 11.37 μVK ^-1^, which produced a *PF*=6.32 μWK ^-2^m ^-1^.

**Figure 2.**
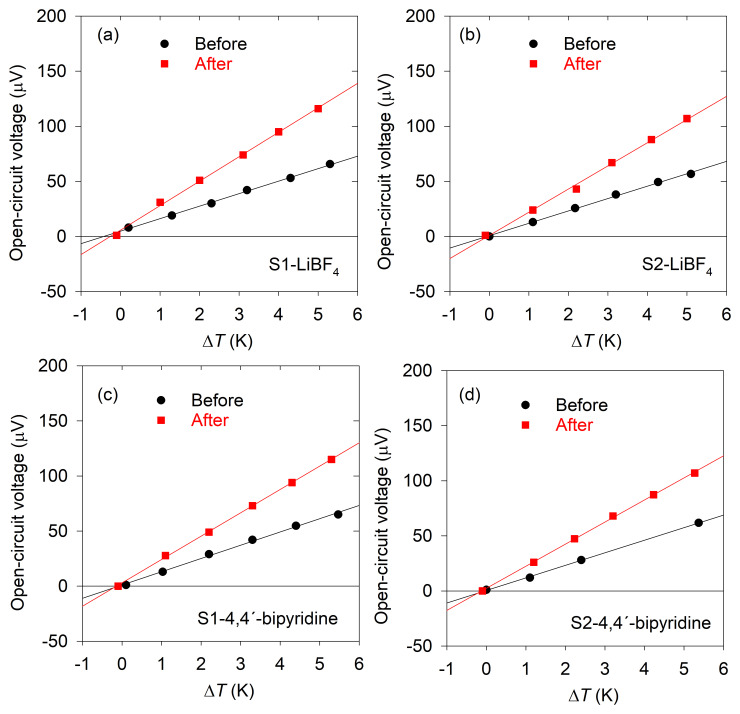
Seebeck coefficient. Open-circuit voltage vs temperature difference curves for the determination of the Seebeck coefficient before and after the treatments. Lines correspond to the linear fits.

**Figure 3.**
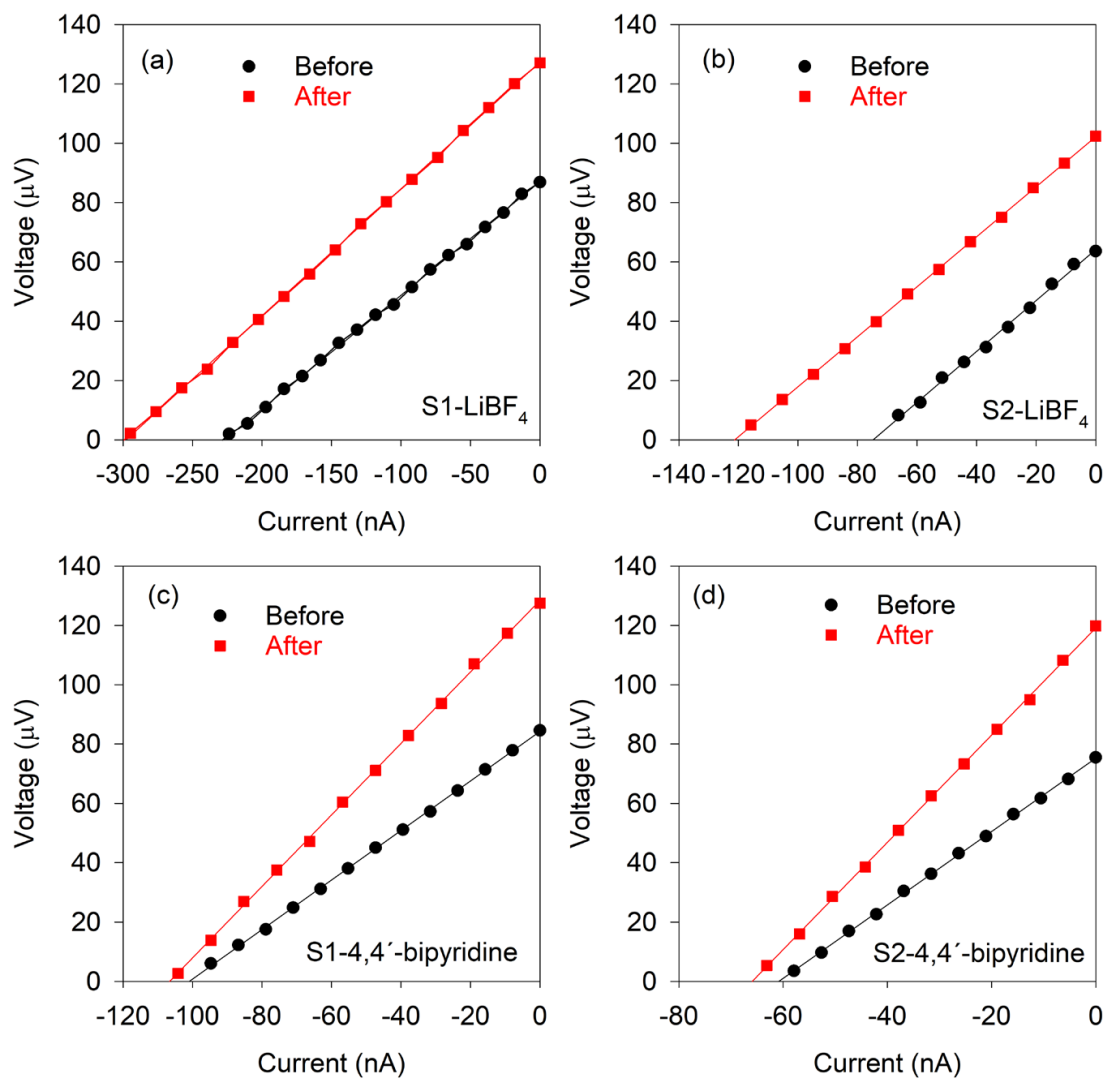
Electrical resistance. Voltage vs current curves for the determination of the electrical resistance before and after the treatments. Lines correspond to the linear fits.

To analyze the effect of the treatments with LiBF _4_ and 4,4'-bipyridine on the TE properties, Seebeck coefficient and electrical resistance measurements were carried out for two different PEDOT:PSS samples for each compound (S1-LiBF _4_, S2-LiBF _4_ and S1-4,4'-bipyridine and S2-4,4'-bipyridine). The results obtained are shown in Figure 2 and Figure 3 and are summarized in Table 1. In addition to these four systems, we also evaluated a sample treated with only the solvent 3-MPN (S1-3MPN) to discard its possible influence on the TE properties (see results in Table 1).

**Table 1.** Power factor variations. Seebeck coefficient, electrical resistance, and their variations, of PEDOT:PSS films before and after the different treatments. Also, the *PF* ratio after and before the treatments is shown.

	Seebeck coefficient (μV/K)			Electrical resistance (kΩ)			*PF*_after_/ *PF*_before_
	Before	After	Variation (%)	Before	After	Variation (%)	
S1-LiBF _4_	11.30	22.20	96.46	0.38	0.42	11.77	3.45
S2-LiBF _4_	11.25	21.07	87.39	0.86	0.84	-3.00	3.62
S1-4,4'-bipyridine	12.04	21.17	75.88	0.82	1.20	46.32	2.12
S2-4,4'-bipyridine	11.58	20.04	73.05	1.22	1.80	47.24	2.05
S1-3MPN	15.92	16.99	6.72	0.39	0.39	0.00	1.13

For the LiBF _4_ systems, it can be observed from Table 1 that there is a significant increase (91.92% average) in the value of the Seebeck coefficient, reaching ca. 21 μVK ^-1^, and a slight increase (4.38% average) in the electrical resistance, leading to a remarkable average *PF* improvement of 3.53 times. It should be noted that this improvement is underestimated because not all the area of the polymer was treated, as the solution did not completely cover the film, but only a certain central area, as shown in Figure 1a.

In the case of 4,4'-bipyridine, it can be seen that there is a large increase (74.46% average) in the Seebeck coefficient, reaching again values of ca. 21 μVK ^-1^, with a substantial rise (46.78% average) in the electrical resistance, leading to a large average *PF* enhancement of 2.08 times. On the other hand, it can be observed from Table 1 that the solvent used in the treatments (3-MPN) had a small influence on *S* (only 6.72% increase) and produced no change in *R*. Hence, LiBF _4_ and 4,4'-bipyridine were the compounds causing the variations in the *PF*.

Considering the initial *PF* value (6.32 μWK ^-2^m ^-1^) and the average improvements obtained, the final *PF* values reached after the treatments can be estimated for LiBF _4_ and 4,4'-bipyridine. This estimation leads to the values of 22.31 and 13.15 μWK ^-2^m ^-1^, respectively, which are close to the values reported for some of the strategies employed to improve the PEDOT:PSS TE performance ^19– 22^.

**Figure 4.**
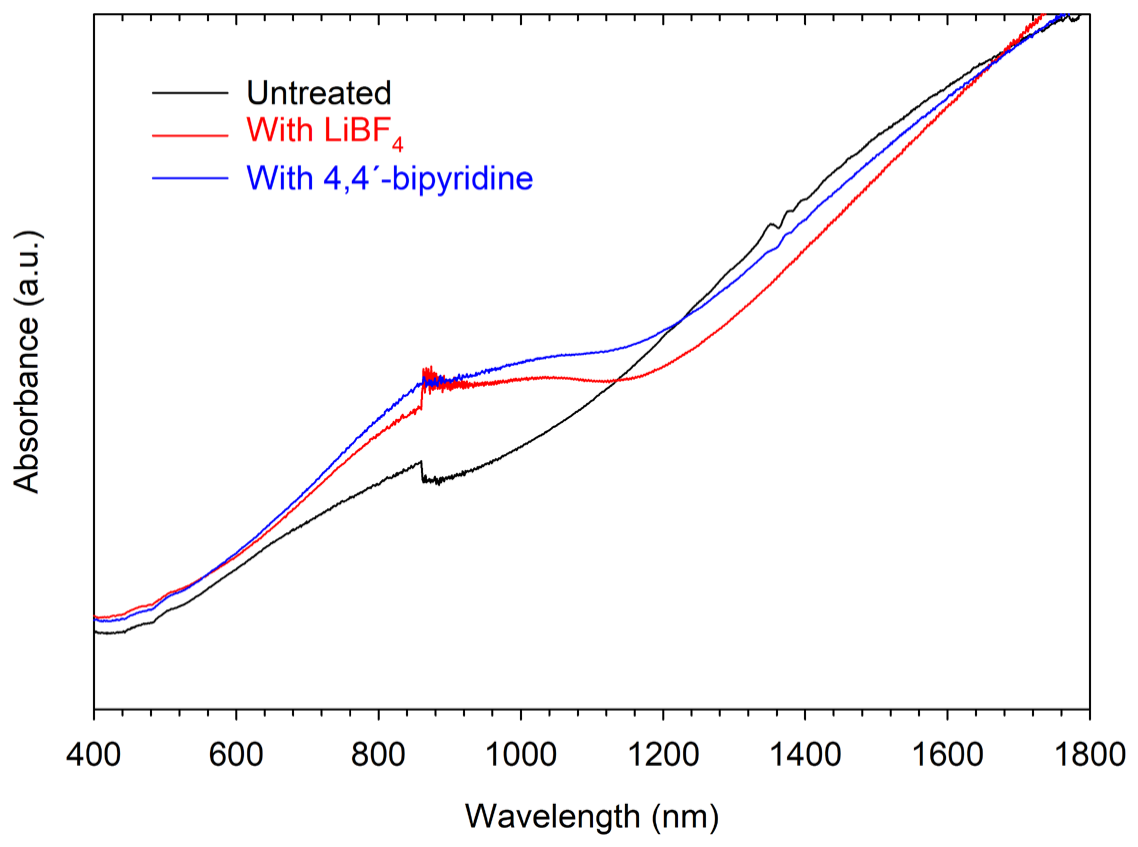
UV-vis-NIR. Absorption spectra of an untreated sample, and samples treated with LiBF _4_ (S1-LiBF _4_) and with 4,4'-bipyridine (S1-4,4'-bipyridine).

To investigate the reasons behind the *PF* enhancement, UV-vis-NIR and Raman spectroscopy measurements were performed before and after the treatments in some of the samples listed in Table 1. The UV-vis-NIR spectra of an untreated sample and the samples treated with LiBF _4_ and 4,4'-bipyridine are shown in Figure 4. It can be observed that for both samples treated with LiBF _4_ and 4,4'-bipyridine, a shoulder appears due to an absorption band centered around 900 nm that is not present in the untreated sample. This band is typically associated with neutral PEDOT segments, indicating a reduction in the concentration of polaronic and bipolaronic states within the polymer matrix, evidencing a dedoping process ^23^.

**Figure 5.**
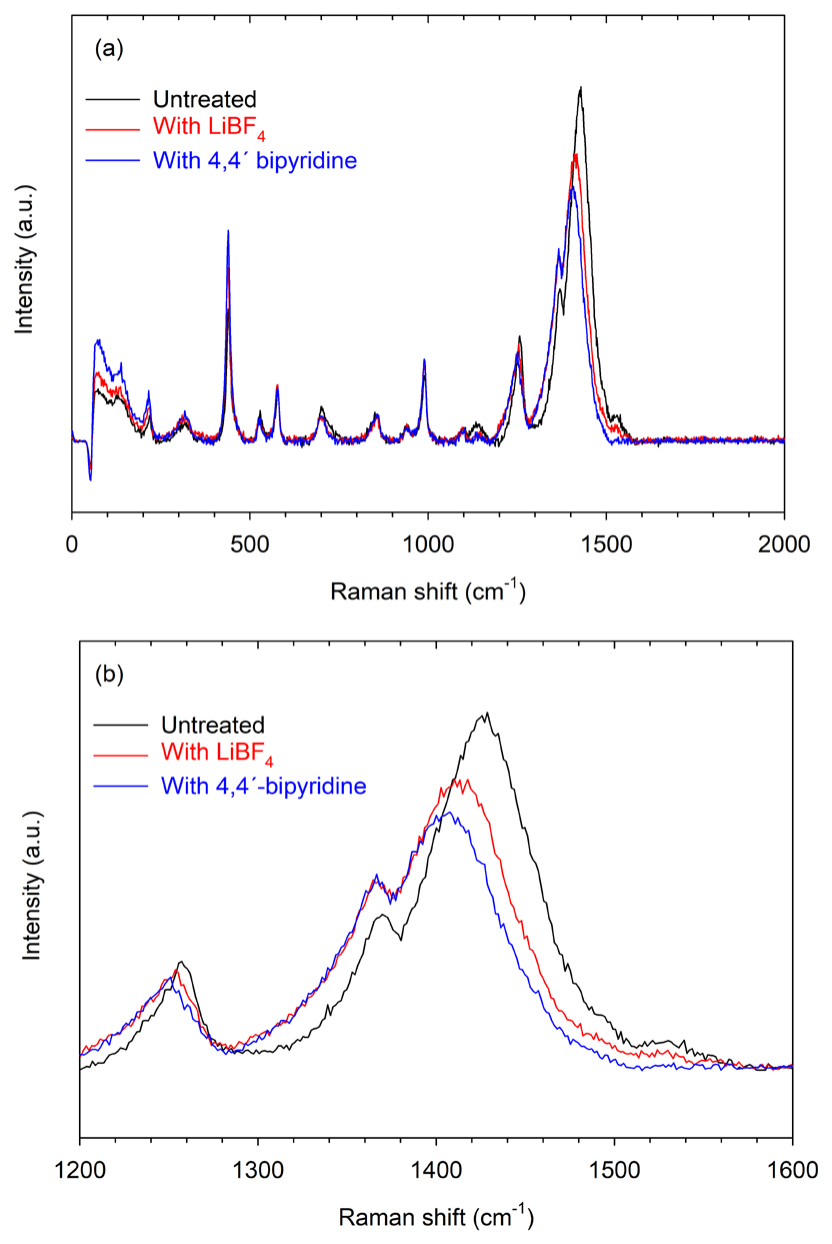
Raman spectra. ( **a**) Untreated sample and samples treated with LiBF _4_ (S1-LiBF _4_) and with 4,4'-bipyridine (S1-4,4'-bipyridine). ( **b**) Magnification of the region of the spectra from 1200 cm ^-1^ to 1600 cm ^-1^.

On the other hand, the Raman spectra of the same samples that were analyzed by UV-vis-NIR spectroscopy are shown in Figure 5. Similar responses were observed in all cases ( Figure 5a), except in the region from 1200 cm ^-1^ to 1600 cm ^-1^ ( Figure 5b). In this region, the Raman bands between 1380 cm ^-1^ and 1500 cm ^-1^ are related to the C _α_=C _β_ stretching vibrations of neutral PEDOT ^16^. It can be seen that the peak of this band is at 1428.79 cm ^-1^ for the untreated PEDOT:PSS film, and shifts to 1413.30 cm ^-1^ and 1406.11 cm ^-1^ for the samples treated with LiBF _4_ and 4,4'-bipyridine, respectively. This redshift is a hallmark of dedoping ^24^. Thus, both UV-Vis-NIR and Raman analyses confirmed an increase in the Seebeck coefficient, attributed to a de-doping mechanism induced by the treatments.

**Figure 6.**
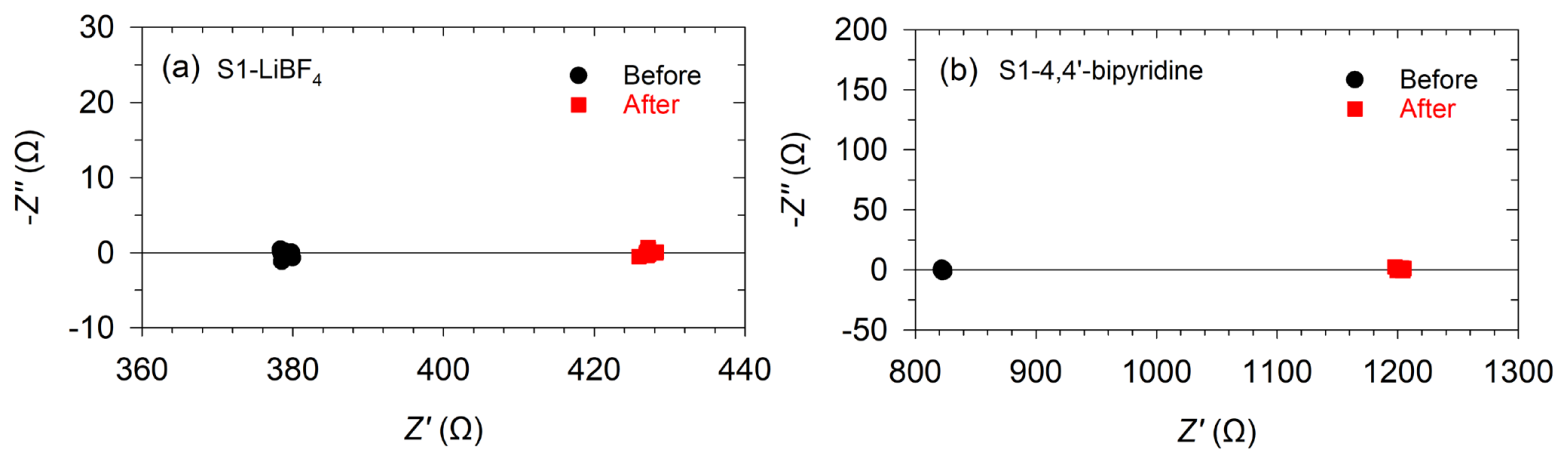
Impedance spectra. Impedance response for PEDOT:PSS films before and after the treatments with ( **a**) LiBF _4_ (S1- LiBF _4_), and ( **b**) 4,4'-bipyridine (S1-4,4'-bipyridine).

Apart from the UV-vis-NIR and Raman analyses, impedance spectroscopy experiments were carried out for S1-LiBF _4_ and S1-4,4'-bipyridine to determine if differences existed in the processes governing these systems before and after the treatments. Figure 6 shows the obtained impedance spectra. They show points lying around certain real impedance ( *Z´*) values in all cases, and no other features appear after both treatments, such as semicircles, capacitive vertical rises, or diffusion (Warburg-like) trends. This indicates that, in all cases, an ohmic resistance (intercept with the *Z'* axis) is the dominant mechanism. As shown in Figure 6, the ohmic resistance increased from 378 to 427 Ω for S1-LiBF _4_ and from 825 to 1203 Ω for S1-4,4'-bipyridine. These variations, in good agreement with the results from Table 1, are coherent with a dedoping process, since this process basically decreases the carrier concentration in the polymer and hence modifies its electrical conductivity.

However, the remarkably small ohmic resistance variation for the LiBF _4_ treatment does not completely match the dedoping process identified by UV-vis-NIR and Raman spectroscopies. Hence, another process might influence carrier transport, which can possibly be a morphological effect on the films.

## Conclusions

In this study, we analyzed the variation in the TE power factor of PEDOT:PSS films after their treatment with two different solutions: (i) a 0.1M solution of LiBF _4_ in 3-methoxypropionitrile and (ii) a 0.1M solution of 4,4'-bipyridine in the same solvent. It was found that after the treatment with LiBF _4_, the films experienced an average 91.92% increase in their Seebeck coefficient and only an average 4.38% increase in their electrical resistance, leading to an at least 3.53 times average power factor improvement with respect to the initial value of 6.32 μWK ^-2^m ^-1^. This is a remarkable result because it is not common to obtain a large increase in the Seebeck coefficient with almost no variation in the electrical conductivity in a single treatment. In the case of 4,4'-bipyridine, it was found a 74.46% increase in the Seebeck coefficient and an average 46.78% rise in the electrical resistance resulting in an at least 2.08 times average power factor improvement. UV-vis-NIR and Raman spectroscopy analyses showed changes corresponding to a dedoping effect for both treatments, which explains the significant increase in the Seebeck coefficient in both cases and the electrical resistance growth in the 4,4'-bipyridine. However, for LiBF _4_, the dedoping effect did not directly produce a significant increase in the electrical resistance, which could be due to the existence of morphological effects in the film after the treatment, which facilitate charge transport. Finally, in order to identify the processes governing the TE performance of the films after the treatments, impedance spectroscopy measurements were performed, showing an ohmic response in all cases (before and after the treatment), so no additional processes appeared due to the treatments. In conclusion, we report two new treatments that can effectively increase the power factor of PEDOT:PSS films to values similar to those achieved by other existing approaches in the literature.

## Ethics and consent

Ethical approval and consent were not required.

## Data Availability

Zenodo: Raw data for the article entitled "Improving the thermoelectric power factor of PEDOT:PSS with 4,4'-bipyridine and LiBF4", https://doi.org/10.5281/zenodo.15775021 ^25^ This project contains the following underlying data: RawData-PEDOT PSS.xlsx: This file contains the raw data for Figure 3 to Figure 6. Data are available under the terms of the Creative Commons Attribution 4.0 (CC-BY 4.0).
