# Supplementary material for: Improving the thermoelectric power factor of PEDOT:PSS with 4,4'-bipyridine and LiBF 4
Source: Open Res Eur. 2025 Aug 4;5:214. [Version 1] doi: 10.12688/openreseurope.20654.1 (PMC12405852; doi:10.12688/openreseurope.20654.1)
Supplement: Peer review history

# Reviewer report: Reviewer response for version 1

**DOI:** 10.21956/openreseurope.22344.r58396

**Published:** 2025-09-02

**Authors:** Zelin Sun

**Affiliations:** 1 The Hong Kong Polytechnic University Shenzhen Research Institute, Shenzhen, Guangdong, China

This article primarily investigates the enhancement of thermoelectric properties of PEDOT:PSS through the use of 4,4'-bipyridine and LiBF4. The authors explore the effects of these two different treatments on the Seebeck coefficient, electrical resistance, and power factor of PEDOT:PSS, demonstrating improvements in thermoelectric power factor with both treatments.

 While the research results are promising, I recommend further exploration of the microstructural changes in the material post-treatment in the discussion. Specifically, utilizing characterization techniques such as X-ray diffraction or SEM could validate whether there are alterations in the crystal structure or morphology of the material after treatment. This would help deepen the understanding of the mechanisms behind the observed changes in thermoelectric performance and provide more concrete guidance for future research.

Is the study design appropriate and does the work have academic merit?

Partly

Is the work clearly and accurately presented and does it cite the current literature?

Yes

If applicable, is the statistical analysis and its interpretation appropriate?

Yes

Are all the source data underlying the results available to ensure full reproducibility?

Yes

Are the conclusions drawn adequately supported by the results?

Yes

Are sufficient details of methods and analysis provided to allow replication by others?

Yes

Reviewer Expertise:

OTE

I confirm that I have read this submission and believe that I have an appropriate level of expertise to confirm that it is of an acceptable scientific standard.

# Reviewer report: Reviewer response for version 1

**DOI:** 10.21956/openreseurope.22344.r58394

**Published:** 2025-09-02

**Authors:** Marcelo Augusto Malagutti

**Affiliations:** 1 University of Trento, Trento, Italy

The present work demonstrates the improvement of thermoelectric power factor pf PEDPT:PSS by the use of 4,4'-bipyridine and LiBF4 solvents. The results and explanation of the obtained improvement are sound and thoroughly explained. The article is highly recommended for indexing after minor enhancements:

 1) The authors mainly focus on the thermoelectric performance of the material. However, since IV plots are available in Figure 3, it would be interesting if the authors plot the power output (multiplying I x V) of the devices and their respective maximum power output in Table1.

 2) Could the authors also compare the current performance with inorganic materials to highlight the advantages of using organic PEDOT:PSS in this case?

Is the study design appropriate and does the work have academic merit?

Yes

Is the work clearly and accurately presented and does it cite the current literature?

Yes

If applicable, is the statistical analysis and its interpretation appropriate?

Yes

Are all the source data underlying the results available to ensure full reproducibility?

Yes

Are the conclusions drawn adequately supported by the results?

Yes

Are sufficient details of methods and analysis provided to allow replication by others?

Yes

Reviewer Expertise:

thermoelectricity

I confirm that I have read this submission and believe that I have an appropriate level of expertise to confirm that it is of an acceptable scientific standard.

# Reviewer report: Reviewer response for version 1

**DOI:** 10.21956/openreseurope.22344.r57719

**Published:** 2025-08-20

**Authors:** Xiaolei Shi

**Affiliations:** 1 Queensland University of Technology, Brisbane, Queensland, Australia

The authors introduced an improvement in the thermoelectric power factor of PEDOT: PSS using 4,4'-bipyridine and LiBF4. I have carefully read the manuscript and found it to be an interesting work with promising results, and the structure and logic are well-organized. However, this work should only be indexed after revisions, since there are still unclear points in the current manuscript. My comments listed below may help the authors further improve their work:

 1.           The current abstract needs to be improved to claim the innovations of this work, such as why this work is timely and unique, that is, why it is distinguished from previous works. Please highlight your work more concisely and targeted.

 2.           In the introduction and background parts, the authors should concisely summarize the most significant progress of thermoelectric materials and their applications, especially for PEDOT: PSS-based thermoelectrics.

 3.           The authors should clearly explain how they determine the error bars when required.

 4.           The concept of improving the thermoelectric performance of PEDOT: PSS through secondary doping, dedoping, and solvent treatments has already been extensively reported in the literature (e.g., acid/base treatments, reducing agents, ionic liquids). This work merely applies the same approach to 4,4'-bipyridine and LiBF4 solutions, without providing a clear scientific rationale for their uniqueness in mechanism or effect. Moreover, the manuscript claims that the effect of LiBF4 treatment may be related to “morphological changes,” but no substantial morphological characterizations (e.g., AFM, SEM, GIWAXS) are provided, making the interpretation overly speculative. Overall, the work lacks an in-depth analysis of new mechanisms.

 5.           The reported increase in Seebeck coefficient is limited (~12 → ~21 μV/K). Although the power factor enhancement (2–3 times) shows some effect, the absolute values (ca. 13–22 μW·K-2·m-1) remain at a low level. Compared with recent advances in organic thermoelectric materials (where PF values can reach several hundreds or even thousands of μW·K-2·m-1), the improvement is clearly insufficient. Furthermore, no demonstration of device performance or flexible applications is provided, with the study remaining confined to thin-film characterization, which limits practical significance.

 6.           The manuscript relies only on UV-vis-NIR, Raman, and impedance spectroscopy to explain the observed changes, and the conclusions remain largely at the conventional “dedoping” level. There is a lack of deeper evidence concerning carrier transport mechanisms, band structure, or microstructural morphology. In addition, the reproducibility and statistical reliability of the comparative treatments are inadequate, as the number of data points is small and the conclusions are not strongly supported.

 7.           Although the literature review section provides some coverage, it fails to clearly analyze the differences between this work and existing high-level studies and thus does not convincingly highlight the unique contribution of the manuscript. Given the lack of novel material design, distinctive mechanistic insights, or significant performance breakthroughs, the actual academic impact of this work is limited.

 8.           There are also issues with clarity and logic in the writing.

 9.           Some outlooks should be added before the conclusion, such as the future direction for practical applications.

Is the study design appropriate and does the work have academic merit?

Partly

Is the work clearly and accurately presented and does it cite the current literature?

Partly

If applicable, is the statistical analysis and its interpretation appropriate?

Partly

Are all the source data underlying the results available to ensure full reproducibility?

Partly

Are the conclusions drawn adequately supported by the results?

Partly

Are sufficient details of methods and analysis provided to allow replication by others?

Partly

Reviewer Expertise:

Thermoelectrics

I confirm that I have read this submission and believe that I have an appropriate level of expertise to state that I do not consider it to be of an acceptable scientific standard, for reasons outlined above.
